# A conversation with Elias Zerhouni

**DOI:** 10.1172/JCI180284

**Published:** 2024-04-01

**Authors:** Ushma S. Neill

Elias Zerhouni has lived lives in academia, government, and the pharmaceutical sector. Currently professor emeritus at Johns Hopkins University and vice chairman and president of OPKO Health, Zerhouni (Figure 1) originally trained as a radiologist, focusing his research on CT- and MRI-based imaging methods to diagnose cancer and cardiovascular diseases. He notably served as the director of the US National Institutes of Health from 2002 to 2008 and later as president of Global Research & Development at Sanofi. For the full interview, see www.jci.org/videos/cgms

*JCI*: What were you like as a child?

Zerhouni: My childhood was characterized by strong education and the sea. I was born in Algeria in North Africa near the capital of Algiers in a very beautiful small village on the beach. My father was a teacher of mathematics and physics in middle school, and my mother was self-educated. She went to school for three years, and then she was thrown out of school by the French colonialists. While I was growing up, it was a time of war: liberation of Algeria from France. I had a very checkered early education with a lot of interruptions because of the war. I had to self-educate a lot, and my father really played a big role in that. I had six brothers, and my father wanted all of us to become engineers, scientists, or physicists, not doctors or lawyers. He thought that doctors and lawyers were not the smartest people in the world.

*JCI*: You were a competitive swimmer?

Zerhouni: Going to the sea every day, you become a good swimmer. When I went to high school, I was on the school’s team. That is where I met my wife. I ended up on the Algerian national team for a short while, but I couldn’t keep up with the studying and the competition. I had wanted to go to the Olympics.

*JCI*: What was your path towards medical school?

Zerhouni: After high school, I worked in a construction lab. I did some testing for concrete and steel, and that’s where I thought I would end up: being a developer or building bridges and roads. During my time in university, I knew I’d have to develop my math skills for eventual construction engineering jobs, so I went to the mathematics branch and I didn’t like it. I thought it was inhuman and dry.

I had an uncle who was a radiologist who recommended that I go into medicine. I realized that in my country, people were poor and tuberculosis was everywhere, and I thought I’d be more relevant treating people than building bridges. My switch to medicine was a big problem for my father, who thought that doctors were stupid: they learned things by rote and there was no science in it. It’s true that medicine at that time was not seen as a leading-edge intellectual activity, at least in my country. It was seen more as a business. You opened an office and you saw a lot of people; you made some money. I was eventually able to convince my father, and I switched from mathematics to medicine at the University of Algiers.

*JCI*: How is it that you ended up on the path that brought you to the US and Johns Hopkins for your radiology residency?

Zerhouni: The random event here was that my radiologist uncle showed me the first CT scan. It was the first image ever obtained by Sir Godfrey Hounsfield, who ended up getting a Nobel Prize for that. My uncle explained the image to me, telling me about the x-ray tube that turns around combined with a computational process and an algorithm that takes the data to reconstruct the image. We could for the first time see the brain, tumors, and ventricles. I fell in love with that idea because at that time I was getting bored with medicine. My father had been right: there was a tremendous amount of rote learning. I thought maybe I could marry medicine on one end and physics and mathematics and computing on the other end.

Unfortunately for me, there was no CT scanner in Algeria. The recommendation was to go to France, the UK, Sweden, or the US to get trained. That led me to take the equivalency exam for the US. I could have gone to France, but my mother said no, as, “They abused us for 130 years; they’ll abuse you another 20.” That’s why I ended up going to the US even though I didn’t speak English at the time. My medical school dean had trained at the NIH, and he knew the dean at Hopkins. And that was it; that’s what started it.

I was only supposed to stay for a few months, learn, and go back because they wanted to build a university hospital in Algeria. Algeria and the US were close at that time, but they evolved in different ways. Algeria did not build the university hospital; it became a military government that became unstable and no longer allowed the return of people like me. In the US, on the other hand, when I started working with scientists and physicians at Johns Hopkins, it clicked. They liked what I was doing, I liked what they were doing, and then there was a residency position that opened up and they asked me to stay.

*JCI*: It sounds like it was not only a boom time for the field of radiology, but also for you personally.

Zerhouni: Yeah, it was. At the time, radiology was not a forefront specialty, but it was the first one that truly brought computerized imaging into medicine. If you have a background in physical sciences, mathematics, and then you like biology, and then you come to an institution like Hopkins where the goal was really to better understand biology in a noninvasive way, combining those three, you have a perfect situation at a time when the wave of computerized medicine was growing.

I was lucky because the wave of computerized medicine happened at the time I joined Hopkins. Everything before that was analog, observational, and empirical, so extracting biological information with the technologies of the day became my central research driver.

My first work was to differentiate benign tumors from malignant tumors in the lung because we knew that calcium content was different between them. We could look at CT numbers and recompute the quantity and the density. At the time, many patients were operated on for lung nodules, which in the majority were benign.

The same thing happened in MRI. When people were looking at the heart beating, they couldn’t measure it because of movement. I came up with a technique called tagging, where with radio frequency, you can impose a pattern on the heart that you can follow as the heart is contracting and measure it with fidelity.

*JCI*: You rose through the ranks of leadership at Hopkins, so were you surprised to be approached to lead the NIH?

Zerhouni: It was a total surprise; I had no inkling whatsoever nor any ambition to become NIH director. In 1995, I became chairman of my department. At that time, there was a crisis at Hopkins; very quickly after that, I was asked to be the executive vice dean because I was telling the dean that Hopkins was in trouble financially, that we had these siloed departments, and that there was no way to bring science together. In a university, if you complain too much you end up being asked to do the job.

Soon after that, Rick Klausner’s [head of the National Cancer Institute] office called me, as he wanted to do strategic planning for the NCI around imaging. At that time, there was no Imaging Institute. I declined to participate, as I had a bad feeling about the NIH being open to all areas of science because all my grants at the intersection of cancer, imaging, and computation had been declined.

Klausner called me back, personally. He said, “I don’t know you, but nobody turns down the NCI director when asked to do a plan like this.” When I told him my problems with the NCI, he asked me to trust that he was truly trying to make changes. So I told him I was willing to do it under one condition: at the end he had to say either yes or no, but not maybe. He gave me carte blanche. That was my first real high-quality interaction with the NIH. Rick was extremely smart and empowered people.

Then in 1999, Harold Varmus [then president and CEO of Memorial Sloan Kettering] called me upon the recommendation of Hopkins’ cancer center director Marty Abeloff to help review the future of radiology and imaging at MSK. I repeated what I’d said to Rick: I’d do it, but he would then need to say yes or no, but not maybe. That led to being on the larger scientific advisory board for MSK, and I was able to widen my perspective from a siloed disciplinary perspective to a much wider understanding of what was driving science. Through that, I connected to the advisory council at NCI. I started to know what the issues were.

People kept knocking on the door, but I was very busy with my department at Hopkins and bringing what I thought was an era of change to the school. I wanted to step back and enjoy my position a little bit. I was totally surprised when I got a call from the White House; I thought they were calling me to talk me into leading the Imaging Institute, but they were calling for the job of NIH director. It was a total surprise.

*JCI*: Are there particular things that you accomplished as NIH director that you remain proud of or on the contrary look back on with difficulty?

Zerhouni: When I was approached to become the NIH director, I wondered why. It was a high-risk proposition for them and for me: I didn’t have the pedigree to be an NIH director. I’m not a molecular biologist. I didn’t have a Nobel Prize. I’m not even in biological sciences. I’m in radiology for God’s sake, a biomedical engineer.

At the time, the federal administration had committed to double the budget. But we had no clue what needed to be done other than doubling the money on the same programs. I did the NIH Roadmap for Medical Research, so that Congress would know where we were going and why. It included a means by which we would have a common fund for initiatives that really could only be done by NIH, not by any one institute.

I was also proud to establish the Pioneer Awards and the New Innovator Awards. Now, the negative things... conflict of interest.

*JCI*: How did you know that it was time to leave? And how did you make the decision about your next step?

Zerhouni: Seven years is long enough in any one job. I thought I had reached a cruising altitude and things were going well. There was no longer the sense that NIH needed to be on the examination table by Congress. Once Congress reauthorizes you, they leave you alone for a good long time. There was strong bipartisan support for NIH. I was able, despite the stem cell controversy, to keep NIH above politics. Essentially, it became boring. I didn’t want to stay another eight years under another president, so I decided to step down before the election of 2008 because I think that’s the elegant way to do it. When I left the NIH, I was asked to consider some presidencies or executive deanships, and I really didn’t have it in me.

At the time, we had created a program called Grand Challenges in Global Health between NIH and the Gates Foundation that I thought was terrific. The Gates Foundation invited me to help run that program, and so I did that while I was also presidential envoy for President Obama.

Those jobs didn’t pay very well, so I thought I’d consult. The CEO of the French company Sanofi approached me related to problems with productivity in R&D. Over the year period that I was consulting, I fell in love with it. Anyway, after a year of that, I had designed a strategic plan for them, and the CEO asked me to take it over and make it happen. That’s how I ended up taking the job. Not that I wanted to. For me, it was the dark side. And then I realized I had been prejudiced. These scientists are terrific in pharma. It has been great.

*JCI*: If you could not be a physician or a scientist, what other career do you think would have captivated you?

Zerhouni: I’m happy with the career I’ve had and the life I’ve had. The thing that breaks my heart is the inequality in the world. Perhaps I could have pursued something in global affairs, trying to break economic inequalities. When I go back to Algeria, I can’t help but feel like I betrayed them in some ways and didn’t do what I could have done. So maybe I could have done more in the field of international equality, political and economic equality.

## Figures and Tables

**Figure 1:**
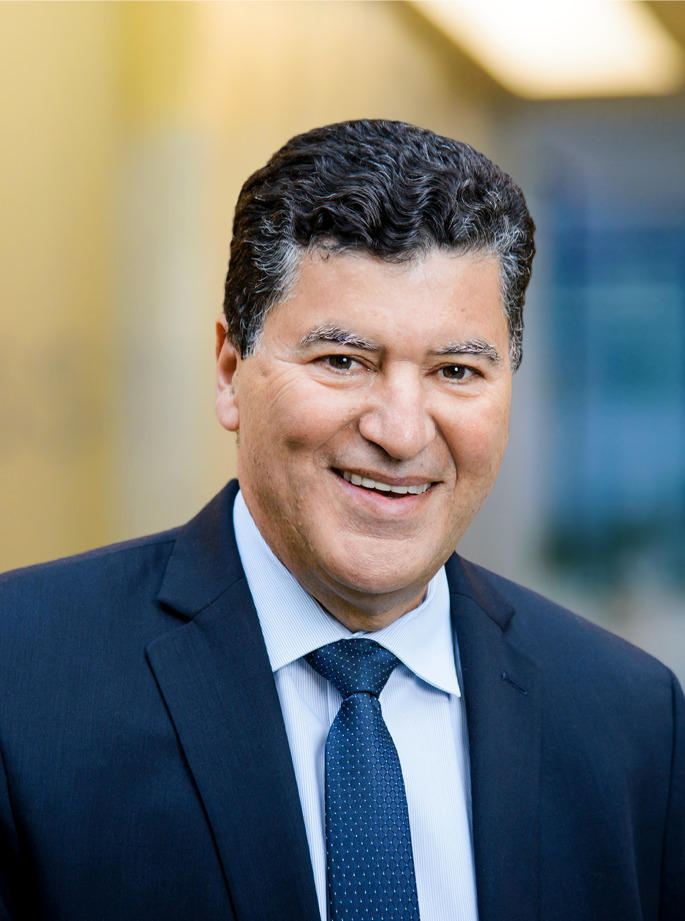
Elias Zerhouni.

## Version 1 04/01/2024

Electronic publication

